# Paediatric face mask for adult ventilation in presence of nasal tumours

**DOI:** 10.4103/0019-5049.79877

**Published:** 2011

**Authors:** Rajeev Sharma

**Affiliations:** Department of Anaesthesia, ESI Hospital, Rohini, New Delhi, India

Sir,

I read with great interest the article by Drs Sethi *et al*. describing the use of a paediatric face mask for mask ventilation in an adult patient with nasal tumour.[1] I congratulate the authors for their improvisation. I have used a Rendell-Baker-Soucek mask for mask-to-stoma ventilation with success.[2] I feel that some aspects of the described method need to be discussed.

Firstly, a closer inspection of the mouth and the neck of the patient in the figure reveals that, in fact, an oropharyngeal airway had already been inserted into the oral cavity for mask ventilation; but this has not been mentioned by the authors.

Second point to be observed and discussed is the need to hold the mask using both hands in order to achieve a tight seal. This necessitates the help of an assistant.

Considering the above two aspects of the technique, I suggest that apart from a laryngeal mask airway, a cuffed oropharyngeal airway (COPA) would be a better alternative, both in terms of efficacy as well as feasibility, than using an oropharyngeal airway along with a paediatric face mask. This is because the COPA can be directly connected to a breathing circuit without the need of a face mask to ventilate. Further, an assistant would not be required.

Another aspect is that many times these tumours are fragile and highly vascular, and desperate attempts at mask ventilation even using small paediatric masks can cause trauma on trivial pressure. Therefore, one has to be very cautious in those cases.
